# Case report: A novel case of parental mosaicism in *SMC1A* gene causes inherited Cornelia de Lange syndrome

**DOI:** 10.3389/fgene.2022.993064

**Published:** 2022-09-28

**Authors:** Marta Gil-Salvador, Ana Latorre-Pellicer, Cristina Lucia-Campos, María Arnedo, María Teresa Darnaude, Aránzazu Díaz de Bustamante, Rebeca Villares, Carmen Palma Milla, Beatriz Puisac, Antonio Musio, Feliciano J. Ramos, Juan Pié

**Affiliations:** ^1^ Unit of Clinical Genetics and Functional Genomics, Department of Pharmacology and Physiology, School of Medicine, CIBERER and IIS-Aragon, University of Zaragoza, Zaragoza, Spain; ^2^ Unit of Genetics, University Hospital of Móstoles, Madrid, Spain; ^3^ Neuropediatrics, University Hospital of Móstoles, Madrid, Spain; ^4^ Service of Genetics, University Hospital “12 de Octubre”, Madrid, Spain; ^5^ Institute for Biomedical Technologies (ITB), National Research Council (CNR), Pisa, Italy; ^6^ Unit of Clinical Genetics, Service of Paediatrics, Department of Paediatrics, University Hospital “Lozano Blesa”, School of Medicine, CIBERER and IIS-Aragon, University of Zaragoza, Zaragoza, Spain

**Keywords:** Cornelia de Lange syndrome, *SMC1A*, parental mosaicism, deep-sequencing, X-linked, genetic counseling, case report

## Abstract

Ultimate advances in genetic technologies have permitted the detection of transmitted cases of congenital diseases due to parental gonadosomatic mosaicism. Regarding Cornelia de Lange syndrome (CdLS), up to date, only a few cases are known to follow this inheritance pattern. However, the high prevalence of somatic mosaicism recently reported in this syndrome (∼13%), together with the disparity observed in tissue distribution of the causal variant, suggests that its prevalence in this disorder could be underestimated. Here, we report a new case of parental gonadosomatic mosaicism in *SMC1A* gene that causes inherited CdLS, in which the mother of the patient carries the causative variant in very low allele frequencies in buccal swab and blood. While the affected child presents with typical CdLS phenotype, his mother does not show any clinical manifestations. As regards *SMC1A*, the difficulty of clinical identification of carrier females has been already recognized, as well as the gender differences observed in CdLS expressivity when the causal variant is found in this gene. Currently, the use of DNA deep-sequencing techniques is highly recommended when it comes to molecular diagnosis of patients, as well as in co-segregation studies. These enable us to uncover gonadosomatic mosaic events in asymptomatic or oligosymptomatic parents that had been overlooked so far, which might have great implications regarding genetic counseling for recurrence risk.

## Introduction

Cornelia de Lange syndrome (CdLS, OMIM #122470, #300590, #610759, #614701, #300882) is a rare, dominant genetic disorder with an estimated incidence of 1 in 10,000–30,000 live births worldwide. It is classically characterized by growth retardation, distinctive dysmorphic facial features, hirsutism, intellectual disability and limb reduction defects (Kline et al., 2018). Its widely variable clinical presentation is likely related to its genetic heterogeneity. In around 60%–70% of clinically diagnosed individuals, a heterozygous loss-of-function pathogenic variant can be found in the cohesin loading factor *NIPBL,* while other seven genes related to the cohesin complex (*SMC1A, SMC3, RAD21*, *HDAC8, BRD4, ANKRD11,* and *MAU2*) are estimated to account for 5%–10% of cases of CdLS (Krantz et al., 2004; Musio et al., 2006; Deardorff et al., 2012a, 2012b; Ramos et al., 2015; Pié et al., 2016; Parenti et al., 2020).

Originally, this syndrome, as most dominant genetic disorders, was thought to be caused by *de novo* pathogenic variants in one of the causative genes (Kline et al., 2018). Nevertheless, unexpected transmitted cases resulted from parental gonadosomatic mosaicism are arising over the last few years in many genetic diseases, including CdLS, principally due to the enhancements in molecular diagnostic techniques, namely, next-generation sequencing (NGS) of DNA, that allows the detection of variants with very low allele frequencies (Campbell et al., 2014).

This approach could be especially relevant regarding CdLS. In this disorder, although a number of familial cases have been reported (Gillis et al., 2004; Krantz et al., 2004; Slavin et al., 2012), only exceptionally, the presence of the causative variant could be confirmed in blood-derived DNA of the parents (Niu et al., 2006; Deardorff et al., 2007; Slavin et al., 2012; Krawczynska et al., 2018; Masciadri et al., 2018). The reason could fairly lie either in the lack of sensitiveness of the detection method or in the presence of the variant strictly in some germinal cells. However, the high prevalence of somatic mosaicism recently identified in CdLS patients, together with the disparity observed in tissue distribution of the pathogenic variant (Latorre-Pellicer et al., 2021), suggests that we could be overlooking some cases of gonadosomatic mosaicism in parents, which, in case of detection, could have great impact on genetic counseling of affected families.

Here we present a new case of parental gonadosomatic mosaicism in *SMC1A* gene that causes inherited CdLS, in which the unaffected mother carries the causative variant in very low allele frequencies in buccal swab and blood. To our knowledge, there is only one CdLS causative variant in *SMC1A* reported thus far as inherited due to parental gonadosomatic mosaicism, being remarkable that the same variant was also found in another familial case, while its presence could never be confirmed in parents’ blood (Deardorff et al., 2007).

## Case description

We report on a 13 month-old male patient, first child of a non-consanguineous healthy couple, born at 38 weeks and 5 days gestational age with no complications during pregnancy, although he presented with late-onset intrauterine growth restriction (IUGR). His birth length (47.5 cm, −1.4 SD), body weight (2.505 kg, −1.89 SD) and head circumference (32 cm, −1.57 SD) were all within normal ranges for gender and age.

At the age of 3 months the infant was transferred to the Neuropediatric Clinic due to hypertonia and dysmorphic facial features. Physical examination revealed microcephaly (HP:0000252), synophrys (HP:0000664), long eyelashes (HP:0000527), low-set ears (HP:0000369), depressed nasal root (HP: 0005280), thin upper vermilion (HP:0000219), receding forehead (HP: 0000340), low posterior hairline (HP: 0002162), and partial bilateral syndactyly in second and third toes (HP:0004691). All these clinical findings were consistent with the diagnosis of CdLS in the patient. After neurological work-up, structural anomalies such as hypoplasia of the cerebellum (HP:0001321) and corpus callosum (HP:0002079), megacisterna magna (HP:0002280) and a possible delay in the brain myelination were detected.

**FIGURE 1:**
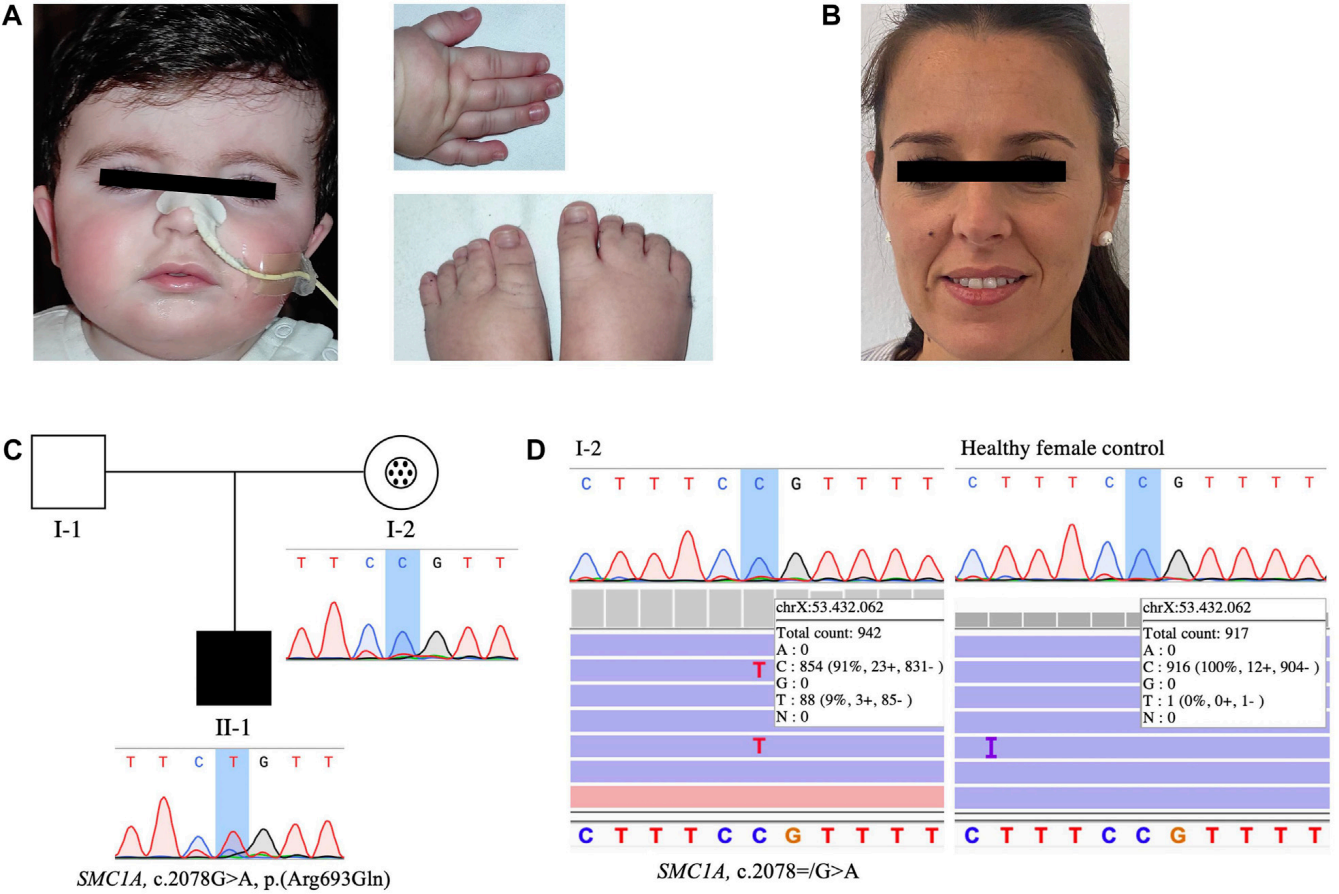
Clinical picture, pedigree of the family and sequencing results of the mother’s buccal swab DNA. **(A)** Facial features, right hand and feet of the affected patient. **(B)** Face of the mosaic carrier mother. **(C)** Pedigree of the family showing Mendelian transmission of the *SMC1A* variant and the different genotypes present in mother and son: black symbol represents the affected patient with heterozygous pathogenic variant (II-1). In individual I-2, the circle in the center of the symbol indicates asymptomatic carrier, and mosaicism condition is highlighted with a black dots pattern inside. Sanger sequencing results of the targeted *SMC1A* variant [NM_006306.3:c.2078G>A, p. (Arg693Gln)] analyzed in blood-derived DNA of the patient and his mother are shown below their respective representative symbols. The non-affected father (I-1) is indicated as an open symbol. **(D)** Sanger chromatogram and Integrative Genomics Viewer (IGV) view of sequencing results of the targeted *SMC1A* variant (NM_006306.3:c.2078 = /G>A) in patient’s mother (left) and a healthy female control (right) buccal swab-derived DNA.

A thorough revaluation was done by our clinical geneticist at 13 months of age. While the measurements of height, body weight and head circumference remained normal, physical examination at that time revealed brachycephaly (HP:0000248), micrognathia (HP:0000347), mild arched eyebrows (HP:0002253) and palate (HP:0000218), and mild dental diastema (HP:0000699). The patient presented additional typical features of CdLS including small hands (HP:0200055), mild hirsutism (HP:0002230), cryptorchidism (HP:0000028), gastroesophageal reflux (HP:0002020), feeding difficulties (HP:0008872) and psychomotor delay (HP:0001249). All these findings confirmed the CdLS diagnosis in the patient, who had a clinical score of 10 according with the recent CdLS diagnosis statement (Kline et al., 2018). The phenotype of the patient is shown in Figure 1A, that was subjected to an additional analysis with the Face2Gene application (Latorre-Pellicer et al., 2020), in which CdLS was the first choice syndrome with a high gestalt level.

Both parents were also evaluated clinically and by using Face2Gene technology and none of them showed any signs or symptoms of CdLS or any other related disorder (Figures 1B,C).

## Genetic analyses

Given the suspicion of a developmental disorder, a 180 k CGH-Array was performed in the patient at the age of 5 months and was negative for any possible genetic alteration that may explain his clinical picture. With the aim of reaching a molecular diagnosis, exome sequencing analysis filtered for the genes related to CdLS was done following xGen Exome Panel v1.0 (Integrated DNA Technologies) kit instructions in the Illumina NextSeq 550 System (RRID:SCR_016381) according to the manufacturer’s protocol. As a result, a potential disease-causing variant was identified in *SMC1A* gene [*SMC1A*:NM_006306.3:c.2078G>A:p. (Arg693Gln)], detected with an allele frequency of 100% and 53x sequencing coverage. This variant is reported on the dbSNP database (rs587784408), and has been classified as pathogenic in ClinVar (RRID:SCR_006169) and Leiden Open Variation Database (LOVD) (RRID:SCR_006566). It has been previously described in literature in a CdLS female patient (Gervasini et al., 2013), and another that affects the same aminoacidic position (p.Arg693Gly), which is actually greatly evolutionarily conserved, has been also reported as deleterious (Liu et al., 2009). rs587784408 is not registered in Genome Aggregation Database (genomAD) (RRID:SCR_014964) population frequency database, and is predicted to be deleterious according to all *in silico* predictive algorithms applied [SIFT (RRID:SCR_012813, https://sift.bii.a-star.edu.sg/): 0.0, PolyPhen: Polymorphism Phenotyping (RRID:SCR_013189, http://genetics.bwh.harvard.edu/pph2/): 1.0]. Thus, this variant was classified as pathogenic based on AMP/ACMG guidelines for variant classification (Richards et al., 2015).

In order to investigate the presumed *de novo* origin of the variant, familial co-segregation studies were performed by Sanger sequencing as a first approach. The primer sequences used are presented in Supplementary Table S1. Remarkably, the chromatogram obtained from SnapGene Viewer (Dotmatics) after Sanger sequencing of DNA from mother’s peripheral blood resulted inconclusive (Figure 1C), and a more comprehensive study of a suspected mosaicism condition was done by testing another tissue sample through deeper sequencing techniques. Thus, a posterior analysis in our laboratory using a custom gene panel was performed as described previously to analyse mother’s DNA isolated from buccal swab (Latorre-Pellicer et al., 2021). This included *SMC1A* with very high sequencing depth, and the analyses of the sequencing results using Ion Torrent Suite (Thermo Fisher Scientific), Ion Reporter (Thermo Fisher Scientific) and Integrative Genomics Viewer (IGV) (RRID:SCR_011793) (Broad Institute) softwares confirmed the presence of the pathogenic variant with very low allele frequency (AAF 9.34%) in the mentioned patient’s mother sample. Deep sequencing yielded a coverage of 942 reads of the targeted genomic position (Figure 1D), which allowed the detection of the variant of interest in such a low mosaic level. Sanger sequencing of the mother’s buccal swab-derived DNA was also carried out, and once again, suggested a possible scenario of genetic mosaicism (Figure 1D), but could not anyway ensure so. These results were contrasted with an additional analysis of a buccal swab sample from a healthy female control, verifying that the detection of the variant with an AAF so low was accurate and did not result from intrinsic errors of the panel (Figure 1D). Finally, the foregoing findings indicated that the variant had been maternally transmitted.

The pedigree of the family (Figure 1C) shows the Mendelian transmission of the variant and the different genetic scenarios present in mother and son, as well as the absence of any clinical manifestations in the patient’s mother (Figure 1B).

## Discussion

As a rule, with regard to dominant genetic disorders, causal variants are understood to occur spontaneously during meiosis, resulting in a mutated sperm or egg. Nevertheless, transmitted cases in these diseases due to gonadosomatic parental mosaicism might be more common than currently appreciated (Campbell et al., 2014).

During the last decade, the increasing technical capabilities of genomic technologies have permitted the detection of variants with very low allele frequencies, elucidating several cases of somatic mosaicism in a number of genetic disorders. Notably, when the mosaicism condition is revealed in a parent of an affected child, the recurrence risk of such disease might substantially increase, which has a relevant effect in familial counseling about future pregnancies. As a proof of principle, we report on a maternally transmitted case of CdLS caused by a pathogenic variant in *SMC1A* gene, thought to be originally *de novo*.

*SMC1A* is an X-linked gene that accounts for about 5% of CdLS cases, in which patients present with similar, but usually milder, clinical features than those with classic CdLS (Borck et al., 2007; Deardorff et al., 2007; Liu et al., 2009; Pié et al., 2010; Rohatgi et al., 2010; Hoppman-Chaney et al., 2012), typically associated with loss-of-function pathogenic variants in *NIPBL*.

The current case refers to an affected child with a phenotype that fits the pattern observed in CdLS patients with variants in *SMC1A* and a mosaic carrier mother who does not show any signs or symptoms of CdLS or any other related disorder. The very low allele frequency found in the mother could explain the absence of clinical manifestations. However, this might not be so obvious in CdLS, considering that mosaic patients usually present clinical features as severe as those with constitutive pathogenic variants (Latorre-Pellicer et al., 2021). Additionally, the arduousness in interpreting clinical presentation for *SMC1A* variant carrier females has been largely recognised (Liu et al., 2009; Gervasini et al., 2013), as well as the gender differences observed in CdLS clinical expressivity when *SMC1A* is the affected gene.

First, the random X inactivation leads female carriers of *SMC1A* pathogenic variants to express both mutant and wild-type alleles, while male carriers express only the mutated one. However, *SMC1A* is reported to partially escape X inactivation (Brown et al., 1995), and, as a consequence, females express twice as much *SMC1A* mRNA as males (Liu et al., 2009) in quite variable allele proportions depending on the individual (Carrel and Willard, 2005). Since CdLS affected females harboring *SMC1A* variants produce a standard total amount of *SMC1A* transcripts compared to control population, dosage sensitivity does not seem critical for the phenotypic effects. Otherwise, it has been proposed that the mutant allele could exert its deleterious effect through a trans-dominant mechanism (Liu et al., 2009). On the other hand, given the attenuated clinical presentation observed for some females with causal variants in this gene compared to males, a compensation favouring the expression of the wild-type *SMC1A* allele has been hypothesized (Musio et al., 2006; Deardorff et al., 2007). While this issue remains to be clarified, it seems feasible that gender, regarding *SMC1A* causal variants, could have an effect in CdLS clinical expressivity.

Only a limited number of cases of CdLS with inherited causative variants in *SMC1A* due to parental mosaicism are reported. Remarkably, the only variant described [*SMC1A*:NM_006306.3: c.1487G>A; p. (Arg496His)] in two sisters with CdLS resulted from a gonadosomatic mosaic father without any clinical presentation, reinforcing the complexity in *SMC1A* variants inheritance pattern and expressivity aforementioned. The same variant was found in another unrelated familial case, and despite it could never be confirmed in parents, its presence in both daughters was consistent with germline mosaicism (Deardorff et al., 2007). Although non CdLS-related, an additional case of parental mosaicism in *SMC1A* was reported in a neurodevelopmental disease, but in this case the mother of the proband was not totally unaffected and suffered from epilepsy (Shu et al., 2021).

Regarding other causative genes, several transmitted CdLS cases through parental mosaicism bias have been reported, especially in the most frequent causal gene of this disorder, *NIPBL* (Gillis et al., 2004; Krantz et al., 2004; Niu et al., 2006; Slavin et al., 2012; Parenti et al., 2016; Krawczynska et al., 2018). Indeed, systematic population-level studies are lacking to assess the contribution of mosaicism to the transmission of CdLS to offspring. However, in regard to postzygotic mosaicism in patients, the prevalence estimated is ∼13%, which entails a high percentage in comparison to other genetic diseases. Moreover, a huge disparity in tissue distribution of mosaic variants has been observed (Latorre-Pellicer et al., 2021). These findings make us think about the possibility that inherited CdLS cases resulted from parental gonadosomatic mosaicism could be more frequent than currently recognized, highlighting the necessity for more accurate co-segregation studies in this disorder.

The identification of low-level somatic mosaicism for clinically relevant variants is challenging. Clinical exome sequencing in patients and accompanied Sanger validation of parental blood-derived DNA are commonly used in routine clinical diagnostics in CdLS, but have limited sensitivity detecting low-frequency variants. During the last few years, the development of high-throughput genomic technologies such as quantitative polymerase chain reaction (qPCR), deep sequencing or droplet digital PCR (ddPCR) has enhanced our capabilities to assess low grade mosaicism (Zemet et al., 2022). As stated above, in our study, a deep targeted panel (>1,000x) was applied allowing the identification of the causal variant with a very low AAF (9.34%) in the mother’s DNA.

Otherwise, the uncertainty about the representative tissues to precisely study mosaicism entails another limitation in its detection. Most genetic tests are performed on blood-derived DNA, but this may not be the best choice sample (Zemet et al., 2022), especially in CdLS, in which great disparity in the distribution of mosaic variants across tissues has been already recognized (Latorre-Pellicer et al., 2021). Thus, the analysis of additional samples besides blood with highly sensitive technologies should be recommended in CdLS co-segregation studies.

Based on previous data, the transmission to offspring of genetic disorders due to apparent *de novo* variants has an average recurrence rate of ∼1% (Rahbari et al., 2016). However, this percentage has proven to be an underestimation, especially for disease-causing variants located on the X chromosome (Helderman-van Den Enden et al., 2009; Lannoy and Hermans, 2020). For mosaic parents, the risk can hypothetically be as high as 50% (Møller et al., 2019). Actually, Rahbari et al. (2016) reported that the transmission to offspring reached 50% when the causal variant was found in greater than 6% of parental blood cells. Therefore, although it has been already demonstrated that the level of somatic mosaicism correlates positively with the overall recurrence risk (Rahbari et al., 2016; Jónsson et al., 2018), the difficulty of calculating the proportion of germinal mutated cells, especially in women, makes the recurrence risk estimation next to impossible. In this context, preimplantation genetic testing (PGT) could be a feasible option for couples who wish to have additional children to avoid intrafamilial recurrence (Zemet et al., 2022).

To conclude, the case herein presented, together with the recent findings about genetic mosaicism in CdLS, underscores the importance of performing diagnostic procedures in patients and families by using DNA deep-sequencing techniques. The identification of gonadosomatic mosaicism in parents could substantially alter recurrence risk estimation, with a significant impact in reproductive genetic counseling.

## Data Availability

The datasets for this article are not publicly available due to concerns regarding participant/patient anonymity. Requests to access the datasets should be directed to the corresponding author.
