# Supplementary material for: Case report: A novel case of parental mosaicism in SMC1A gene causes inherited Cornelia de Lange syndrome
Source: Front Genet. 2022 Sep 28;13:993064. doi: 10.3389/fgene.2022.993064 (PMC9554350; doi:10.3389/fgene.2022.993064)
Supplement: Supplementary file 1 [file Table1.pdf]

Supplementary Table 1: List of primer sequences used for Sanger sequencing.

Gene	Primer sequence (5’-3’)		Annealing temperature (°C)
SMC1A	Forward	CAGGCTCAGTACTGGAGATT	58
	Reverse	AACCTAGGCCAGGAATGTGT	58
