# Supplementary material for: Case report: A novel case of parental mosaicism in SMC1A gene causes inherited Cornelia de Lange syndrome
Source: Front Genet. 2022 Sep 28;13:993064. doi: 10.3389/fgene.2022.993064 (PMC9554350; doi:10.3389/fgene.2022.993064)
Supplement: Supplementary file 2 [file Table1.DOCX]

**Supplementary table 1:** List of primer sequences used for Sanger sequencing.

| **Gene** | **Primer sequence (5’-3’)** | **Annealing temperature (**$\boldsymbol{^{\circ}}$**C)** |
| --- | --- | --- |
| *SMC1A* | Forward CAGGCTCAGTACTGGAGATT | 58 |
|  | Reverse AACCTAGGCCAGGAATGTGT | 58 |
